# TGF-βl Suppresses Inflammation in Cell Therapy for Intervertebral Disc Degeneration

**DOI:** 10.1038/srep13254

**Published:** 2015-08-20

**Authors:** Huilin Yang, Cheng Cao, Chunshen Wu, Chenxi Yuan, Qiaoli Gu, Qing Shi, Jun Zou

**Affiliations:** 1 Department of Orthopaedic Surgery, The First Affiliated Hospital of Soochow University, Suzhou, Jiangsu 215006, China

## Abstract

Recent studies suggest that cell therapy may be an effective way to repair intervertebral disc degeneration. As a strong immune suppressor, TGF-β1 has been shown to inhibit inflammation respond effectively. The objective of this study was to explore the effects of TGF-β1 during bone marrow mesenchymal stem cells-based therapy for disc degeneration. *In vitro* assays demonstrated that co-culturing of nucleus pulposus cells with bone marrow mesenchymal stem cells resulted in significantly higher levels of TGF-βl secretion. This increase inhibited I*κ*B phosphorylation and NF-*κ*B activation, detected by western blot analysis. Meanwhile, in a rabbit model, MRI analysis revealed significant recovery of signal intensity in the degenerative discs of rabbits receiving cells transplantation, than receiving cells treated with a TGF-β1 inhibitor or saline. These findings indicated that enhanced TGF-β1 production recovered the degeneration of intervertebral disc. And also immunohistochemical staining detected enhanced collagen II expression in the rabbits treated with cell transplantation. However, the NF-*κ*B positive cells were significantly less than other two control groups. Thus, cell therapy promoted TGF-β1 expression in nucleus pulposus, leading to anti-inflammatory effects via the inhibition of NF-*κ*B, and the amelioration of disc degradation due to increased expression of collagen II and aggrecan in degenerative intervertebral disc.

Health surveys indicated that the prevalence of low back pain was 15%–45%, and that 70%–80% of the population has experienced at least one episode of low back pain at some point in their lives^1^. The current strategies for treatment, including conservative and surgical treatment, are aimed at merely alleviating the symptoms instead of focusing on eliminating the fundamental causes of the pain, and therefore often lead to recurrences of *in situ* or adjacent disc disease^2^. Consequently, the search for more effective treatments for disc degeneration is a current research hot spot. Recent studies suggest that transplantation of bone marrow mesenchymal stem cells (BMSCs) is an effective method for repairing disc degeneration^3^. Several studies have confirmed that BMSCs can delay degenerative changes in nucleus pulposus cells (NPCs)^4^,^5^,^6^. While the mechanism by which this occurs is not yet clear, a recent study demonstrated that BMSCs can be stimulated to differentiate into nucleus pulposus-like cells *in vitro*^7^. The research conducted by Vadalà demonstrated that BMSCs are also stimulated to secrete several growth factors and cytokines such as transforming growth factor-β1(TGF–β1), platelet-derived growth factor, and those factors can promote tissue repair of NPCs^8^. TGF-β1 is a multifunctional factor that regulates cell growth, adhesion, and differentiation in a wide variety of cell types^9^. It is also a strong immune suppressor^10^. However, inflammatory immune responses play an important role in disc degeneration diseases. And nuclear factor kappaB (NF-*κ*B), central to the pro-inflammatory cascade, is activated in degenerative intervertebral discs. Since the activated NF-*κ*B is associated with TGF-β1 pathway in variety of cellular activities, we hypothesized that transplantation of BMSCs would protect against disc degeneration *in vitro* and vivo, by suppressing NF-*κ*B-dependent signaling through TGF-β1.

## Results

### BMSCs Promote TGF-βl Expression in NPCs

**Figure 1:**
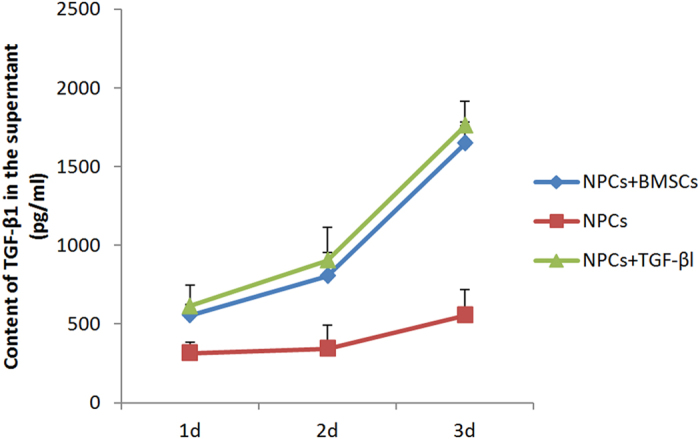
The supernatant media collected from different groups were analyzed for TGF-β1 concentration measured by ELISA. ELISA assay showed that the expression of TGF-β1 in NPCs increased gradually after co-cultured with BMSCs, compared to single culture group. However, there was no significant difference between the co-culture group and the TGF-βl-induced group.

To evaluate the BMSCs’ capability of TGF-βl induction on NPCs, we co-cultured these two cells in six-well Transwell cell culture plates. About 1 × 10^6^ BMSCs were used to coat the bottom chamber of the Transwell system, while 1 × 10^6^ NPCs were added to the upper chamber. About 1 × 10^6^ NPCs treated with recombinant TGF-β1 proteins (at a final concentration of 30 ng/mL) were used as a positive control group, while a single culture containing 1 × 10^6^ NPCs served as the negative control. At 1, 2 and 3 days after co-culture, cells were harvested and seeded in at 1 × 10^6^ cells/well. The supernatants from different populations were then harvested for ELISA experiments. The levels of TGF-βl protein secretion by NPCs increased from 555.16 ± 133.64 to 805.14 ± 212.84 pg/mL between days 1 and 2 of co-culturing with BMSCs, as determined by ELISA analyses. Conversely, there were relatively low concentrations of TGF-βl at days 1 (315.74 ± 71.42 pg/mL) and 2 (342.33 ± 148.49 pg/mL) in the control group (NPCs single culture). At the day 3 time point, there was a significant increase in the levels of TGF-βl secretion by the co-culture group compared with control group (1648.75 ± 155.56 vs. 555.16 ± 133.64 pg/mL, *p* < 0.05). Meanwhile, there was no significant difference in cytokine secretion between the co-culture group and the recombinant TGF-βl-induced group (1761.25 ± 165.46 pg/mL, *p* > 0.05). (Fig. 1).

### Enhanced TGF-β1 from BMSCs Ameliorates NPCs Degradation

**Figure 2:**
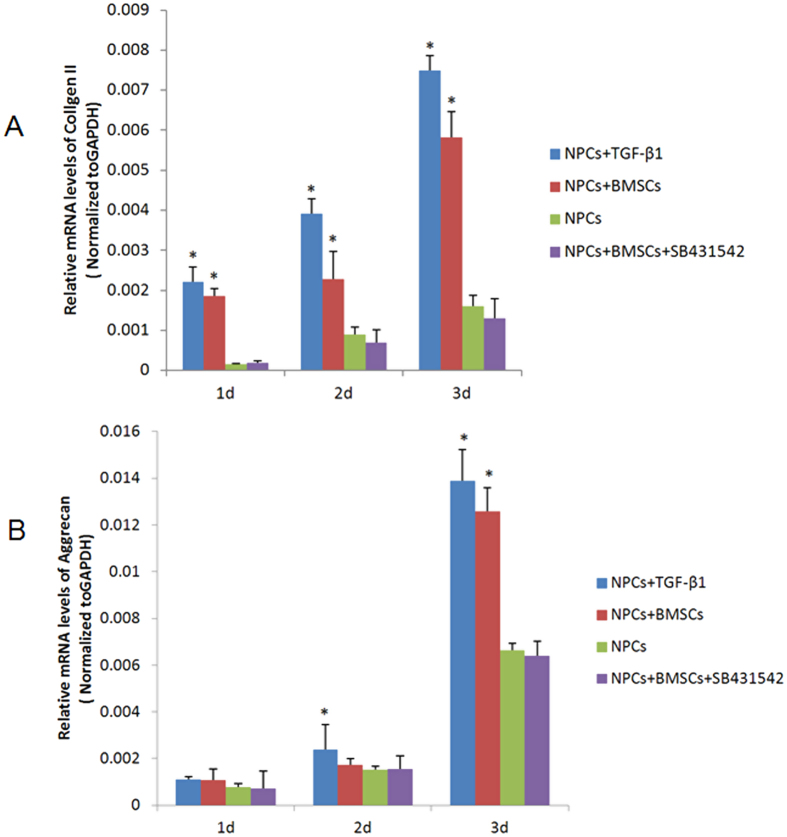
Effect of TGF-β1 on the expressions of chondrogenic genes collagen II and aggrecan *in vitro*. Real-time PCR showed the relative expressions of collagen II and aggrecan were significantly increased in NPCs co-culturing with BMSCs. In contrast, the co-cultured NPCs treated with the TGF-β1 specific inhibitor exhibited stable and low expression levels of mRNA at each time point. (*p < 0.05, compared to NPCs single culture group).

To find clues to protection of BMSCs against nucleus pulposus degradation, we used real-time PCR to investigate the expression of markers of chondrogenesis. Real-time PCR analyses demonstrated that the mRNA expression levels of the chondrogenic genes collagen II and aggrecan were significantly increased in NPCs after 3 days of co-culturing with BMSCs, compared to the single culture control (*p* < 0.05). In contrast, no significant differences in expression were observed between the co-culture group and the group treated with recombinant TGF-βl on day 3 (*p* > 0.05). In contrast, the co-cultured NPCs treated with the TGF-β1 specific inhibitor SB431542 exhibited stable and low expression levels of collagen II and aggrecan mRNA at each time point. No significant differences were observed compared to the NPCs control group at any time point (*p* > 0.05). (Fig. 2).

### TGF-β1 Inhibits I*κ*-B phosphorylation and NF-*κ*B activation

**Figure 3:**
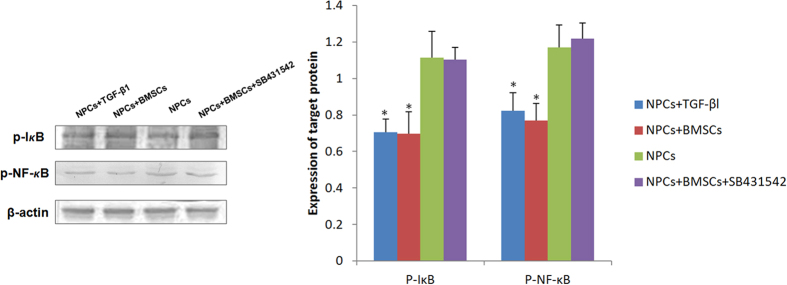
Effect of TGF-β1 on the expression of phospho-I*κ*B and NF-*κ*B protein were detected using western blot. There was a marked decrease in the levels of phospho-I*κ*B and NF-*κ*B in the NPCs co-cultured with BMSCs compared to the NPCs single culture control. The inhibition was attenuated by treatment of the NPCs with a TGF-β1-specific inhibitor, and strengthened by treatment with recombinant TGF-βl protein. (*p < 0.05, compared to NPCs single culture group).

Up-regulated TGF-β1 from BMSCs improved NPCs degradation significantly. In light of these data, we wondered how TGF-β1 is involved in. Therefore, we next analyzed whether the increase of TGF-β1 affects NF-*κ*B pathway. The levels of phospho-I*κ*B and NF-*κ*B were measured by western blot analysis. There was a marked decrease in the levels of phospho-I*κ*B and NF-*κ*B in the NPCs co-cultured with BMSCs compared to the levels in the NPCs single culture control (*p* < 0.05). These findings indicate that NF-*κ*B activation and I*κ*B phosphorylation was suppressed in the co-cultured NPCs. Furthermore, the inhibition of NF-*κ*B activation and I*κ*B phosphorylation was attenuated by treatment of the NPCs with SB431542, and strengthened by treatment with recombinant TGF-βl protein (*p* < 0.05). (Fig. 3).

### TGF-β1 Counteracts Disc Degeneration in a Rabbit Model of BMSCs Transplantation

**Figure 4:**
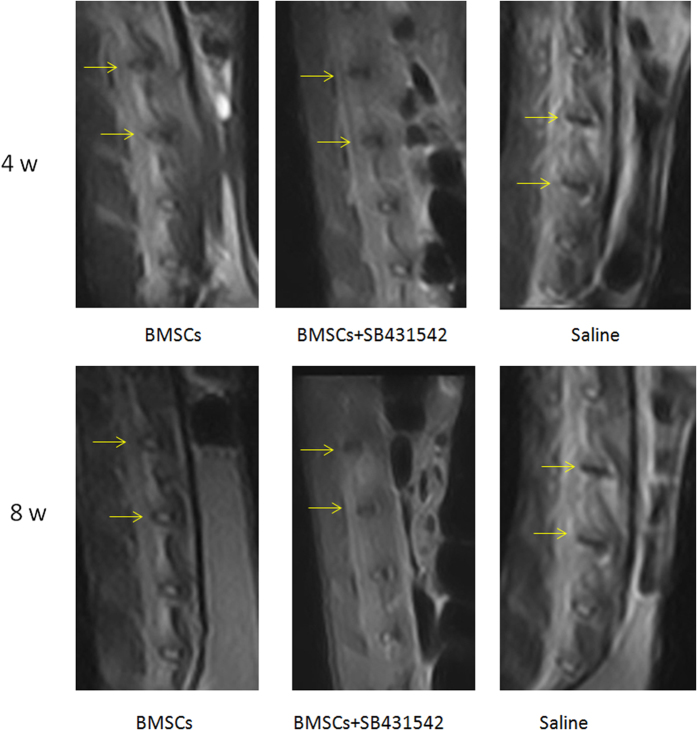
MRI findings after cell transplantation into the rabbit nucleus pulposus. T2 signal intensity in the nucleus pulposus of the BMSCs-injected discs was stronger than the discs of other two control groups.

To evaluated the potential of TGF-β1 *in vivo*, a rabbit model of BMSCs transplantation was demonstrated and assessed. Brief, two consecutive rabbit lumbar intervertebral discs were exposed and punctured using an 18G needle to induce disc degeneration. Four weeks after the initial puncture, either 1 × 10^6^ BMSCs, BMSCs + TGF-βl inhibitor SB431542 or saline only were injected. A sagittal T2-weighted image was obtained to establish the positions of lumbar discs from L4-5 and L5-6. There were significant decreases in the MRI T2-weighted signal intensities shortly after the induction of disc degeneration in the SB431542 and saline groups. Signal intensities were only restored in the BMSCs transplantation group post-transplantation. MRI of the rabbit nucleus pulposus in the BMSCs transplantation group showed stronger signal intensities than those in the control and the TGF-β1 specific inhibitor-treated group at both 4 and 8 weeks after injection. (Fig. 4).

**Figure 5:**
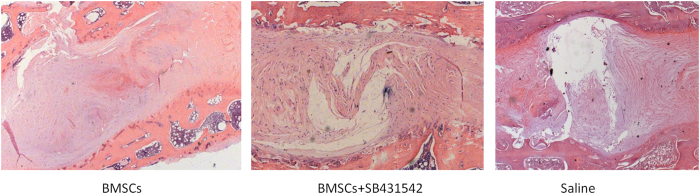
Histological analysis on intervertebral discs by H-E staining (×25). Many nucleus pulposus cells were observed in the BMSCs transplantation group. However, the number of nucleus pulposus cells decreased and were replaced with fibrocartilage tissue in the two control groups.

**Figure 6:**
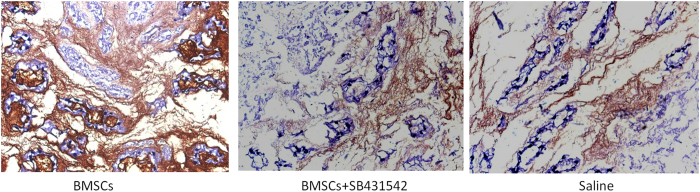
Collagen II immunohistochemical staining (×200). Collagen II, which provides the extracellular matrix framework in cartilage tissue, was detected in the BMSCs transplantation group, but little in the two control groups.

A normal nucleus pulposus contains a large number of nuclear cells. After 4 weeks, there was a decrease in the number of nuclear cells in the nucleus pulposus of the saline and SB431542 groups, and the nucleus pulposus became tissue-like fibrocartilage containing even fewer nuclear cells after 8 weeks. However, in the group receiving BMSCs transplantation, spindle cells formation was observed within the stent after 4 weeks, and there were many nuclear cells outside of the stents. Over the time course, the stent areas slowly decreased in size as the number of cells both within and around the stents declined. (Fig. 5). Collagen II, a typical marker, was detected in the nucleus pulposus tissue in BMSCs transplantation group greatly, but little to no collagen II was expressed in the two control groups. (Fig. 6).

### TGF-β1 Expression Suppresses NF-*κ*B activation *in vivo*

**Figure 7:**
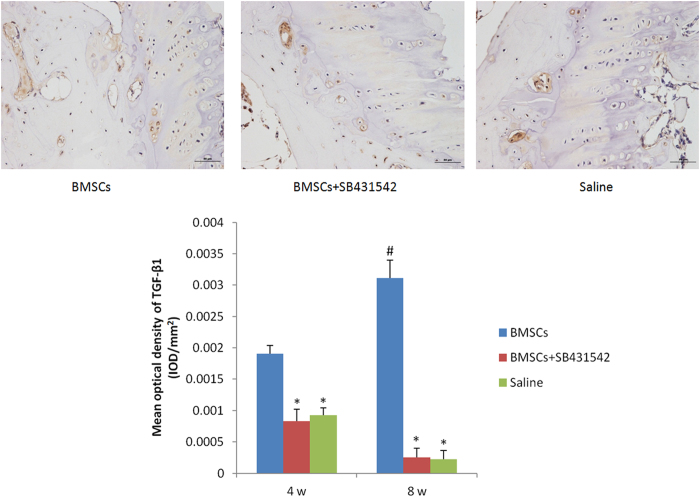
TGF-βl immunohistochemical staining (×200). The expression of TGF-βl was more apparent in the BMSCs transplantation group (8 weeks). Quantification of immunohistochemical staining showed that the BMSCs transplantation had significantly higher mean optical density than the TGF-β1 specific inhibitor-treated and saline group. And the expression of TGF-βl also increased over time significantly. (*p < 0.05, compared to BMSCs transplantation group; #p < 0.05, compared to mean optical density at 4 weeks).

**Figure 8:**
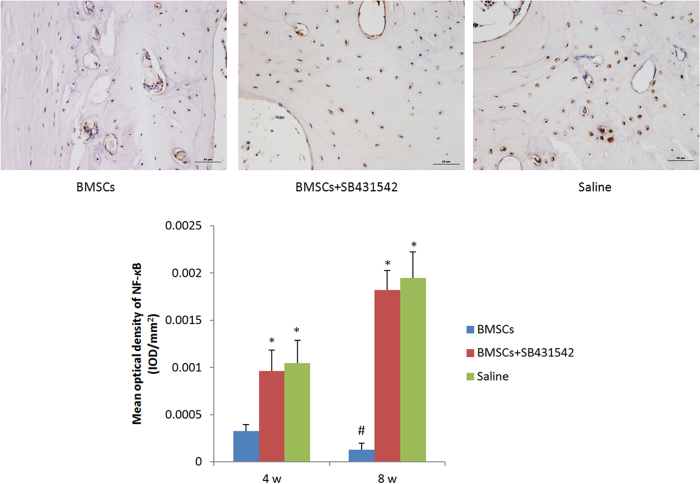
NF-*κ*B immunohistochemical staining (×200). The expression of NF-*κ*B was more apparent in the BMSCs transplantation group (8 weeks). Quantification of immunohistochemical staining showed that the BMSCs transplantation had significantly lower mean optical density than the TGF-β1 specific inhibitor-treated and saline group. And the expression NF-*κ*B also decreased over time significantly. (*p < 0.05, compared to BMSCs transplantation group; #p < 0.05, compared to mean optical density at 4 weeks).

To understand that suppression effects of TGF-βl in NF-*κ*B-dependent inflammation are responsible for the protection against rabbit disc degeneration, we next used immunohistochemistry to detect the expressions of TGF-βl and NF-*κ*B. Immunohistochemical analyses revealed that the expression of TGF-βl increased over time in the BMSCs transplantation group, and also the expression in these discs was significantly higher compared to those detected in the saline and SB431542 groups. (*p* < 0.05) (Fig. 7). However, the expression of NF-*κ*B decreased over time in BMSCs transplantation group. And the expression was significantly lower compared to two control groups. (*p* < 0.05). On the other hand, the expressions of both TGF-βl and NF-*κ*B in SB431542 group was no significant differences when compared with the saline group. (*p* > 0.05) (Fig. 8).

## Discussion

With advances in our understanding of the pathophysiology of disc degeneration, biological treatment techniques through which the degenerative process of disc cells may be delayed or restored, including molecular treatment, gene therapy, and cell therapy, have gained intense scientific interest^11^. In recent years, cell transplantation therapy in particular has provided a new therapeutic strategy for functional recovery of degenerated discs through the restoration of intervertebral disc cells. BMSCs are characterized as having a low immunogenicity, a high self-renewal capacity, and a potential for multilineage differentiation. Indeed, BMSCs were shown to differentiate into a variety of cell types, including osteoblasts, chondrocytes, and fat cells, in particular microenvironments and in the presence of certain cytokines. Because BMSCs are abundant and are easily harvested using relatively simple isolation and culture techniques, these cells have become one of the most extensively studied adult stem cell types^12^. Furthermore, because chondrocytes originate in the mesenchyme, the transplantation and subsequent differentiation of BMSCs into cells expressing NPCs-like phenotypes is an appropriate treatment. Many researches examined the regenerative effect of autologous BMSCs on disc degeneration, and demonstrated BMSCs may induce recovery of disc height and proteoglycan levels in both a rabbit and canine model^3^,^13^,^14^,^15^. However, the mechanism behind this scenario have yet to be defined.

Degeneration of intervertebral discs is a common cause of pain and disability, and is characterized by anatomic, morphologic, and biochemical changes, including altered expression of both matrix metalloproteinases and proinflammatory cytokines^16^. It has been suggested that inflammation may be a factor for explaining the catabolic processes during disc degeneration. The inflammatory cascade associated with disc degeneration is composed of a highly organized but complex sequence of events mediated by various molecules, including degraded matrix enzymes and cytokines^17^. Elevated levels of molecular mediators of inflammation have been described in pathologic disc tissue, with the levels of these mediators increasing with grade of degeneration^18^,^19^.

The TGF-β family of cytokines includes the isoforms TGF-β1, 2, and 3. Although homologous in structure and performing similar activities in many bioassay systems, the TGF-β isoforms are encoded by different genes, exhibit distinct tissue distribution patterns, and perform distinct biological activities within the body^20^. TGF-β1 is one of the most extensively studied growth-regulatory factors. Peng *et al.* assayed the TGF-β1 contents in both normal and degenerative human intervertebral disc tissues by immunohistochemistry, and revealed that the TGF-β1 levels in the normal disc tissues were significantly higher than those in the degenerative tissues. These findings suggested that TGF-β1 plays a role in disc aging and degeneration^21^. Disc degeneration is also an inflammatory disorder associated with significant morbidity and mortality. TGF-β1, as an immune suppressor, has been shown to inhibit NF-*κ*B dependent inflammatory responses in multiple cell types^22^,^23^,^24^. NF-*κ*B is a nuclear protein factor possessing transcriptional regulatory activity, and is present in the cells of various tissues. I*κ*B is an immediate early gene located downstream of NF-*κ*B that functions to inactivate this molecule. The expression of I*κ*B is induced at high levels in response to NF-*κ*B activation, thereby providing a mechanism to control NF-*κ*B signaling^25^. Our laboratory has preliminarily reported that BMSCs might delay NPCs matrix degeneration potentially through the concomitant upregulation of TGF-β1 and the downregulation of NF-*κ*B; however the precise mechanism is not yet present^26^. In this study, we further demonstrated that TGF-β1 can inhibit NF-*κ*B activity through induction of I*κ*B expression as well as through prevention of I*κ*B degradation *via* both *in vitro* transwell system and an *in vivo* rabbit model.

In the co-culture study, we found that BMSCs secrete the cytokine TGF-β1 and exhibit enhanced expression of aggrecan and collagen II. Furthermore, our results indicate that TGF-β1 likely down-regulated NF-*κ*B activity by suppressing I*κ*B phosphorylation. However, in the presence of a TGF-βl inhibitor, BMSCs were unable to suppress I*κ*B inactivation, demonstrating that TGF-βl is critical for suppression of inflammatory cytokines. These findings suggest that cell therapy could provide a protective effect against the inflammatory responses of NPCs via the production of TGF-βl.

Similar results were observed in our rabbit model. Based on MR imaging, high signal intensity of T2 weighted MR imaging reflects the amount of water in the nucleus pulposus. Possibly, cell therapy promoted synthesis of aggrecan, in which negative charged sulfate held water. This water retention was not reduced in the rabbits at 8 weeks. In contrast, intervertebral discs in the TGF-βl inhibitor and control groups preceded a decrease in the water content of the nucleus pulposus.

According to our histological analyses, the effects of cell therapy could be observed as early as 4 weeks. Under high-power microscopy, we identified small chondrocytes within the dense and homogeneous proteoglycan-rich matrix of the rabbit nucleus pulposus. Immunohistochemical analyses demonstrated that the amount of collagen II in the nucleus pulposus was higher in the cell transplantation group than in the TGF-βl inhibitor and control groups. These findings indicate that TGF-βl induced increased production of collagen II, which acted as a frame work in the nucleus pulposus, resulting in the maintenance of disc height and histological features.

A number of studies noted that degradative enzymes and inflammatory cytokines are highly expressed by nucleus pulposus in degenerative intervertebral discs. Each of these molecules results in inflammatory effects. Inhibition of inflammatory cytokine-related genes could provide protective effects against intervertebral disc degeneration. In our study, TGF-βl secretion by BMSCs affected the nucleus pulposus by inhibiting the expression of degradative enzymes and inflammatory cytokines, resulting in maintenance of the intervertebral disc structure. While immunohistochemical analysis detected varying levels of NF-*κ*B at the 4-week time point, the overall levels had dropped by 8 weeks, indicating that there was an association between the inflammatory response and the levels of TGF-βl in the cell transplantation group. However, the level of NF-*κ*B was significantly much more well retained in the TGF-βl inhibitor and saline groups.

## Conclusions

Cell transplantation could prevent intervertebral disc degeneration through a TGF-βl-mediated immune suppressive effect *in vivo*. Co-culture assay *in vitro* revealed that nucleus pulposus cells changed their characteristics by interaction with elevating TGF-βl to inhibit inflammatory response, which suppressed NF-*κ*B activation by protecting I*κ*B phosphorylation and subsequent degradation. Our study provides useful insights into the molecular mechanisms that govern the immune response in intervertebral discs, and suggest the use of intradiscal cell therapy as a potential strategy for treating or preventing disc degeneration diseases.

## Materials and Methods

### Isolation and culture of primary BMSCs

All experimental protocols were approved by Ethics Committee of the First Affiliated Hospital of Soochow University, and carried out in accordance with the approved guidelines. Approximately 1.5 mL of bone marrow was extracted from a New Zealand white rabbit using aseptic techniques. The heparinized bone marrow was diluted in an equal volume of D-Hank’s solution (Gibco, Grand Island, NY), mixed with Percoll separation medium (Sigma, St Louis, MO), and centrifuged at 200 g for 30 min. Cells in the interface above the separation medium were collected and washed twice with phosphate-buffered saline (PBS). After removing the supernatant, the cells were cultured in Dulbecco’s modified Eagle’s medium (DMEM) (Invitrogen, Carlsbad, CA) containing 10% fetal bovine serum (Gibco, Grand Island, NY), 100 mg/L of streptomycin, and 100 U/mL of penicillin in an incubator at 37 °C and 5% CO_2_. The medium was replaced 48 h after the primary inoculation and every 2 days thereafter. Once the cultured cells reached 80% confluence, the cells were digested with 2.5 g/L of trypsin (Invitrogen, Carlsbad, CA) and passaged at a dilution of 1:2.

### Isolation and culture of primary NPCs

Rabbit intervertebral disc was extracted immediately after sacrifice. Under sterile operating conditions, the whole thoracolumbar spine was removed, stripping the front disc attached to the muscles, with D-Hank’s solution (containing 1 g/L streptomycin and penicillin 1,000,000 U/L) rinsed two times, cut fiber ring, remove the gelatinous nucleus pulposus with DMEM/F12 (including 0.1 g/L, streptomycin and penicillin 100,000 U/L) wash 2–3 times after 37 °C type II collagenase solution (Sigma, St Louis, MO) in the 0.25% 15–20 min, suspension with 1,000 rpm under the conditions of centrifugal 8 min, and 1 × 10^5^ cells/mL were used to inoculate 25 mL DMEM/F12 medium and incubated at 37 °C with 5% CO_2_ and saturated humidity.

### Transwell Co-culture

Cells were cultured with DMEM/F12 containing 10% FBS in 6-well Transwell (0.4 μm pore sized) cell culture plates (Corning Life Sciences, Tewksbury, MA). About 1 × 10^6^ BMSCs were used to coat the bottom chamber of the Transwell system, while 1 × 10^6^ NPCs were added to the upper chamber. To clarify the mechanisms of cell therapy, a population of NPCs was treated with the TGF-β1 specific inhibitor SB431542 (Cyagen Biosciences, Santa Clara, CA) at a final concentration of 10 μM for 30 min. Approximately 1 × 10^6^ SB431542-treated NPCs were then placed in the upper chamber of a separate Transwell, and 1 × 10^6^ BMSCs were again placed in the lower chamber.

### Enzyme Linked Immunosorbent Assay (ELISA)

TGF-β1 secretion of different groups were analyzed using ELISA kits (Westang Biotech, Shanghai, China), according to the manufacturer’s instructions. Absorbance measurements for all samples and standards were performed in triplicate, and were read at a wavelength of 450 nm using an ELISA plate reader (Bio-Rad Model 680, Hercules, CA). The TGF-β1 concentration of each sample was determined by extrapolation from a standard curve made out of the standards provided by the manufacturer. All samples and standards were measured in duplicate.

### Real-time PCR

Cells from four groups were collected at 1, 2 and 3 days after co-culture, and the total RNA contents of these cells were extracted using TRIzol reagent (Invitrogen, Carlsbad, CA). The RNA samples were quantified and reverse transcribed into first-strand cDNA. To detect the expression of the osteogenic genes osteocalcin and osteopontin, fluorescence-based quantitative PCR was performed using an ABI Prism 7500 Sequence Detection System (Applied Biosystems, Foster City, CA). Glyceraldehyde-3-phosphate dehydrogenase (GAPDH) expression was used as a reference, and was amplified in parallel with the target sequences. Each reaction was carried out in 10 μL volume, and included 5 μL of SYBR Green mixture, 1 μL of each primer (5 mM), 3 μL of cDNA template (10 mM). The PCR amplification conditions were as follows: 50 °C for 2 min, 95 °C for 10 min, 95 °C for 15 sec, and 60 °C for 1 min for 40 cycles. The upstream and downstream sequences for each primer were as follows:

GAPDH: 5′-AAGGTCGGAGTGAACGGATTTG-3′, 5′-CGTGGGTGGAATCATACTGGAAC-3′;

Collagen II: 5′- GCTCCCAGAACATCACCTACCA-3′, 5′-ACAGTCTTGCCCCACTTACCG-3′;

Aggrecan: 5′-AGGTCGTGGTGAAAGGTGTTGTG-3′, 5′-TGGTGGAAGCCATCCTCGTAG-3′.

### Western Blot Analysis

Cells from each group were harvested after 24 h of co-culturing. Cells were washed three times with PBS and suspended in ice-cold lysis buffer. Lysates were separated by 12% SDS-PAGE and proteins were transferred to nitrocellulose membranes. Membranes were blocked with 1 × TBS-Tween-20 buffer containing 5% milk, and incubated overnight at 4°C in the presence of phospho-I*κ*B and NF-*κ*B specific primary antibodies (Biosynthesis Biotech, Beijing, China). The following day, membranes were washed and incubated with the corresponding peroxidase-conjugated secondary antibodies at room temperature for 1 h. The membranes were then developed using enhanced chemiluminescence, and relative protein concentrations were measured using Quantity One software (Bio-Rad, Hercules, CA). The expression level of β–actin was used as an internal control.

### Experimental animals

30 New Zealand rabbits were randomly divided into three groups. The rabbits were then placed into a lateral prone position and the anterior surfaces of two consecutive lumbar intervertebral discs (L4-5 and L5-6) were exposed via a posterolateral retroperitoneal approach. The nucleus pulposus tissues of the three consecutive lumbar intervertebral discs were punctured using an 18G needle on a 5 ml syringe to induce disc degeneration. Four weeks after the initial puncture, either 1 × 10^6^ BMSCs, BMSCs + TGF-βl inhibitor SB431542 or saline only were injected into the center of the rabbit nucleus pulposus from the contralateral side using a 26-gauge needle with a microsyringe.

### Magnetic resonance imaging (MRI)

MRI was performed on each rabbit at 0, 4 and 8 weeks after the operation to assess the degree of disc degeneration. Sagittal T2-weighted images were obtained using a 0.2 T E-Scan XQ Extremity MRI (Esaote, Genoa, Italy).

### Histological and immunohistochemical analysis

The intervertebral discs were harvested, fixed in 4% paraformaldehyde, decalcified with 10% ethylenediaminetetraacetic acid, and embedded in paraffin using standard procedures. The sections were stained with hematoxylin and eosin (H-E) for evaluation of disc degeneration. For immunohistochemistry detection, sections were incubated with primary antibodies specific to collagen II (Novus Biologicals, Littleton CO), TGF-β1 (Novus Biologicals, Littleton CO), or NF-*κ*B (Biosynthesis Biotech, Beijing, China). The images obtained were captured on an image analysis system and analyzed with the Image Pro Plus 6.0 software (Media Cybernetics, Baltimore, MD, USA). Integrated optical density was calibrated and the area of interest was set. Mean optical density = integrated optical density/area (mm^2^).

### Statistical analysis

All quantitative data are presented as mean ± standard deviation (SD). Statistical analyses were performed using one-way analysis of variance (ANOVA). Difference with p < 0.05 was considered statistically significant.

## Additional Information

**How to cite this article**: Yang, H. *et al.* TGF-βl Suppresses Inflammation in Cell Therapy for Intervertebral Disc Degeneration. *Sci. Rep.* **5**, 13254; doi: 10.1038/srep13254 (2015).
